# Gene Modified CAR-T Cellular Therapy for Hematologic Malignancies

**DOI:** 10.3390/ijms21228655

**Published:** 2020-11-17

**Authors:** Wen-Ying Lin, Hsin-Hui Wang, Yi-Wei Chen, Chun-Fu Lin, Hueng-Chuen Fan, Yi-Yen Lee

**Affiliations:** 1 Department of Internal Medicine, Taipei Veterans General Hospital, Taipei 11217, Taiwan; sincesnow@gmail.com; 2 Department of Pediatrics, Division of Pediatric Immunology and Nephrology, Taipei Veterans General Hospital, Taipei 11217, Taiwan; hhwang@vghtpe.gov.tw; 3 Department of Pediatrics, Faculty of Medicine, School of Medicine, National Yang-Ming University, Taipei 11221, Taiwan; 4 Institute of Emergency and Critical Care Medicine, School of Medicine, National Yang-Ming University, Taipei 11221, Taiwan; 5 Division of Radiation Oncology, Department of Oncology, Taipei Veterans General Hospital, Taipei 11217, Taiwan; chenyw@vghtpe.gov.tw; 6 School of Medicine, National Yang-Ming University, Taipei 11221, Taiwan; cf_lin@vghtpe.gov.tw; 7 Department of Neurosurgery, Neurological Institute, Taipei Veterans General Hospital, Taipei 11217, Taiwan; 8 Department of Pediatrics, Tungs’ Taichung Metroharbor Hospital, Wuchi, Taichung 435403, Taiwan; wylin17@vghtpe.gov.tw; 9 Department of Medical Research, Tungs’ Taichung Metroharbor Hospital, Wuchi, Taichung 435403, Taiwan; 10 Department of Life Sciences, National Chung Hsing University, Taichung 402, Taiwan; 11 Department of Rehabilitation, Jen-Teh Junior College of Medicine, Nursing and Management, Miaoli 356, Taiwan; 12 Division of Pediatric Neurosurgery, Department of Neurosurgery, Neurological Institute, Taipei Veterans General Hospital, Taipei 11217, Taiwan

**Keywords:** acute lymphoblastic leukemia (ALL), diffuse large B cell lymphoma (DLBCL), multiple myeloma (MM), chimeric antigen receptor (CAR)-T cells, gene modified-based cellular platform, immunotherapy

## Abstract

With advances in the understanding of characteristics of molecules, specific antigens on the surface of hematological malignant cells were identified and multiple therapies targeting these antigens as neoplasm treatments were developed. Among them, chimeric antigen receptor (CAR) T-cell therapy, which got United States Food and Drug Administration (FDA) approval for relapsed/refractory (r/r) diffuse large B-cell lymphoma (DLBCL) as well as for recurrent acute lymphoblastic leukemia (ALL) within the past five years, and for r/r mantle cell lymphoma (MCL) this year, represents one of the most rapidly evolving immunotherapies. Nevertheless, its applicability to other hematological malignancies, as well as its efficacy and persistence are fraught with clinical challenges. Currently, more than one thousand clinical trials in CAR T-cell therapy are ongoing and its development is changing rapidly. This review introduces the current status of CAR T-cell therapy in terms of the basic molecular aspects of CAR T-cell therapy, its application in hematological malignancies, adverse reactions during clinical use, remaining challenges, and future utilization.

## 1. Introduction

### 1.1. History of Immunotherapy in Hematological Malignancy 

Hematological malignancies, such as Hodgkin disease, and leukemia, were first described in the 19th century [1]. Since then, numerous regimens of therapies, mostly chemotherapy, were developed over the past two centuries. However, allogeneic hematopoietic stem cell transplantation (allo-HSCT), which transfuses a matched donor’s peripheral blood or bone marrow stem cells to a recipient who has received conditioned chemotherapy to kill off most cancer cells, seems to be the only curative treatment or the regimen milestones in many hematological malignancies [2]. Indeed, allo-HSCT, first performed in 1968, is the precursor of immunotherapies, as it allows immune cells from the donor to recognize and demolish “non-self” cells [3]. Recently, with the discovery of the molecular basis of tumor cells, multiple immunotherapies for cancers including monoclonal antibodies, antibody-drug conjugates, bispecific T-cell engagers, checkpoint inhibitors, and chimeric antigen receptor (CAR) T-cell therapies have evolved and have expeditiously acquired approval from the United States Food and Drug Administration (FDA) [3]. Among them, CAR T-cell therapy for selected hematological malignancies provides a nascent platform for cancer treatments. Herein, we provide an overview of the applications of CAR T-cell therapy to hematologic malignancies, with discussions of its limitations and future perspectives.

### 1.2. Molecular Structure of CAR T-Cell

**Figure 1:**
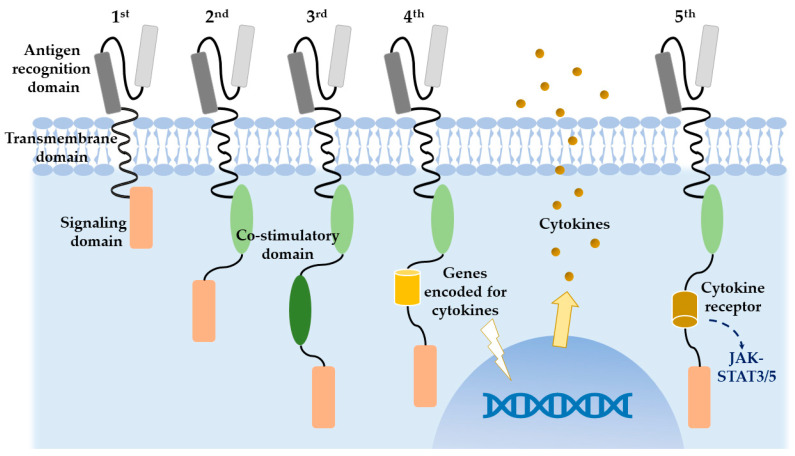
Structures of five generations of CAR T-cell therapy. The extracellular antigen-binding domain typically comprised of variable heavy and light chains to form a single chain variable fragment (scFv) from a monoclonal antibody. The ectodomain is then coupled with the endo-domain through the transmembrane domain. In the first generation, the intracellular domain is typically equipped with CD3ζ of the T-cell receptor. In the second generation, CD28 or 4-1BB is added to the intracellular domain. The modified third generation then contains both. The novel fourth generation contains genes encoded for cytokines for transgenic expression, such as IL-12 and IL18, which can further activate cytokines. The developing fifth generation comprise of IL-2 receptor β-chain domain that further promotes cytokine cassette.

CARs are artificial recombination proteins that contain three main parts—an extracellular antigen-recognition domain, a transmembrane domain, and an intracellular tyrosine containing activation motifs [4,5]. The part outside cell membrane is an antigen-targeting moiety purified from a monoclonal antibody, consisting of a single-chain variable fragment (scFv), a fusion protein of the variable regions of heavy and light chains. Once bound to tumor antigens, it is responsible for triggering T-cell activation and leads to cytokine release, cytolytic degranulation, and T-cell proliferation [6]. As for the intracellular domain linking to extracellular domain through a transmembrane domain, it determines the quality, strength, and persistence of a T-cell response to tumor antigens [7]. Different fragments are incorporated for corresponding malignancies on the outer domains, while the inner domain provides the space for improving the efficacy of CAR T-cell therapies and yields five generations of CARs to date (Figure 1). The initial generation of CARs, whose endodomain contains only CD3-ζ chain or FcεRIγ, supports inadequate T-cell expansion, a short in vivo life span and insufficiently secreted cytokines [8,9,10]. An intracellularly costimulatory domain CD28 [11,12] or 4-1BB [13] was then added to generate the second generation of CARs, which ameliorated T-cell proliferation, response to tumor antigens, and in vivo persistence [14]. To achieve higher potency, CD28 and 4-1BB were combined together, and yielded the third generation [15,16]. As for the fourth generation, in addition to adoptive immunity, interleukin-12 (IL-12) or other cytokines (such as IL-8, 9, 15, and 18) was tethered to the endodomain of the second generation, in an attempt to activate innate immunity at the same time. This manipulation led to the recruitment of tumor infiltrating T-cells (TILs) and natural killer cells that were able to eliminate antigen-negative cancer cells [17]. This amalgamation is termed as T-cell redirected for universal cytokine-mediated killing (TRUCKs) [14]. Activating cytokines not only modifies the tumor microenvironments but also results in prolong activation of CARs and protects T-cell from activation-induced cell death. This finding is currently in its early phase of clinical trials [18]. Recently, fifth generation of CARs was proposed as a product of insertion of an IL-2 receptor β-chain domain, with a binding site for the transcription factor STAT3. This can induce robust cytokine (JAK–STAT3/5) signaling in the targeted tumor tissues and reduce systemic side effects [19], thereby broadening the use of CAR T-cell therapy to a variety of other diseases.

### 1.3. Protocol of CART-Cell Therapy

**Figure 2:**
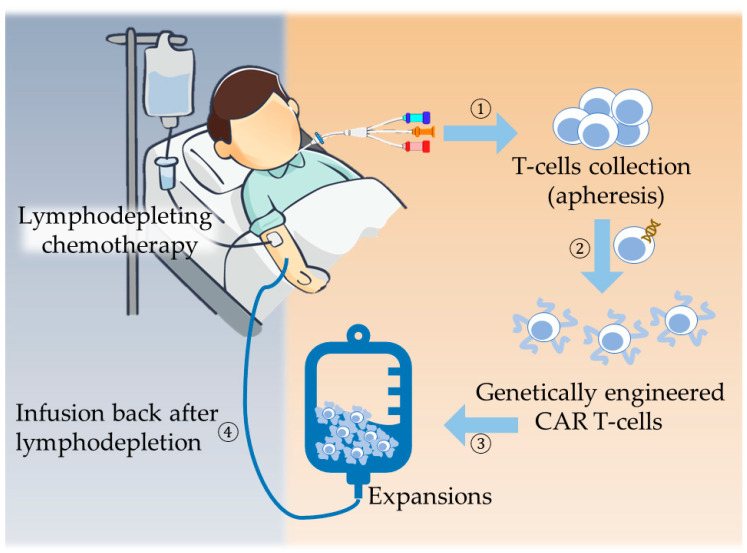
CAR T-cell therapy protocol: (1) T-cell collected from patient’s peripheral blood. (2) Artificial antigen and costimulatory domains are engineered into T-cells. (3) Amplification of CAR T-cells to sufficient amount. (4) Infuse CAR T-cells back into patient’s body after lymphodepletion.

Current regimen of CAR T-cell therapy follows a general protocol (Figure 2)—patient’s T-cells are first collected, purified, and activated with antibodies or antibody-coated beads artificially. This is followed by transduction of CAR molecule into T-cell using transient transfection via lentivirus, retrovirus transduction, or electroporation [20]. The modified CAR T-cells were then multiplied in vitro to a sufficient amount before being infused back into the patient. Prior to the transfusion, the patient receives lymphodepleting chemotherapy, such as various doses of cyclophosphamide alone, fludarabine and cyclophosphamide, pentostatin and cyclophosphamide, bendamustine-based regimens, and several disease-specific regimens determined at physician’s discretion. The addition of lymphodepletion chemotherapy was anecdotally shown to increase persistence of CAR T-cell therapy. No regimen was clearly shown to be superior in terms of efficacy for optimizing CAR T-cell activity, nor is it clear which particular method is more toxic than another [20].

## 2. CAR T-Cell Therapy in Hematological Malignancies

In 2017, two anti-CD19 CAR T-cell products, Tisagenlecleucel and axicabtagene ciloleucel, received FDA approval, respectively. Tisagenlecleucel was approved for multiple relapsed/refractory (r/r) pediatric acute lymphoblastic leukemia (ALL). Moreover, both tisagenlecleucel and axicabtagene ciloleucel could be used for diffusing large B-cell lymphoma (DLBCL) after two or more lines of therapy. In 2020, the FDA further granted the use of brexucabtagene autoleucel, a CD19-directed CAR T-cell therapy for adult patients with r/r mantle cell lymphoma (MCL). Currently, multiple trials are ongoing, and a list of current antigens targeting different hematological malignancies are summarized in Table 1. The following paragraphs discuss the applications and limitations of common hematological malignancies. 

### 2.1. r/r B-Cell Acute Lymphoblastic Leukemia

The response to tisagenlecleucel in patients with r/r B-cell ALL, was first well understood during a phase 1–2a study conducted at the Children’s Hospital of Philadelphia and the University of Pennsylvania [39]. The study involved 60 children and young adults and yielded a complete remission rate of 93% [39]. The 4-year follow-up demonstrated significant disease control without additional therapy needed [39,40]. The ELIANA trial, based on these results, extended the research area to 25 study sites in 11 countries across North America, Europe, Australia, and Asia. The trial revealed 61 out of 75 (81%) patients less than 21-years-old had remission within 3 months, and the event-free survival and overall survival were 50% and 76% at 12 months, respectively [41]. This result promoted FDA’s approval for tisagenlecleucel in young adult with r/r B-cell ALL. Recent update of the ELIANA trial in 2019, under a median follow-up of 24 months, demonstrated ongoing response in 29 patients (45%), with a current maximum duration of 29 months [22]. Further clinical trials using CD19 CAR T-cell therapy in patients with r/r ALL also showed remarkable results, with the complete response rate varying from 67% to 93% [41,42,43,44,45,46,47,48,49,50]. Patients’ quality-of-life after tisagenlecleucel infusion at 3 months also showed dramatic improvement based on a patient-reported questionnaire [51].

However, relapse of CD19 negative clones was detected in up to 20% of patients, post-CAR T-cell therapy [39]. In this situation, CD22 CAR might play a role as anti-CD22 antibody, inotuzumab, which recently received approval for relapsed B-cell ALL [52], and CD22 CAR was reported to induce remissions in patients who were CAR T-cell therapy naïve or with relapse after anti-CD19 CAR T-cell therapy [23]. Furthermore, the studies focused on targeting more than 1 antigen, such as CD19 with either CD20 or CD22, are ongoing [53,54] and might change the paradigm of the r/r ALL treatment.

### 2.2. r/r Large B-Cell Lymphoma

DLBCL is the most common non-Hodgkin’s lymphoma [55]. Despite its noteworthy response to the classic R-CHOP regimen, namely combination of rituximab with cyclophosphamide, doxorubicin, vincristine, and prednisone, up to a 15% refractory rate within 3 months was noted and around 35% patients had a chance of relapse [56]. In patients with primary refractory DLBCL or relapse, receiving autologous transplantation only yielded a complete response rate of 7% per year and a median overall survival of 6.2 months [57], calling for the need of advanced treatments. In a phase 2a, single-center study, the tisagenlecleucel in patients with r/r DLBCL, demonstrated a 50% response rate at 3 months, with 43% having a complete response at 6 months [58]. Based on this result, the JULIET trial was initiated. The update of the trial revealed that the overall response rate was 52%, with 40% of the 93 patients showing complete responses, and 12% showing partial responses [30]. The median overall survival among patients who received an infusion was 12 months [30,59]. Axicabtagene-ciloleucel, another anti-CD19 CAR T-cell therapy, showed 4 out of 7 patients with complete response, and 3 remained in remission at 1 year [60]. A phase 2 trial of ZUMA-1 was then conducted and the response rate was 82%, with a complete response rate of 54% [29], which later led to FDA’s approval of axicabtagene-ciloleucel for r/r DLBCL. The 2-year follow-up data involving 108 r/r LBCL patients, declared overall response rate as 82%, with a 58% complete response [61]. The updated analysis by Locke and colleagues, with a median follow up of 27.1 months, still observed ongoing responses in 39% of patients, with 37% maintaining complete response [62]. The real-world use of axicabtagen-ciloleucel across 17 academic centers in the US also reported an overall response rate of 79% with 50% complete response, consistent with the aforementioned clinical trials. These data led to three ongoing randomized phase III clinical trials in primary refractory or high-risk relapsed DLBCL (NCT03391466, NCT03575351, and NCT03570892). The control arm in each of these studies involved second-line intensive chemotherapy, followed by autologous stem cell transplant. Another CAR T-cell therapy lisocabtagene-maraleucel, also CD19-directed, is currently under investigation in late-stage clinical trials but is not yet approved by the FDA [33]. However, emerging data with high rate of relapse or progressive disease post-CAR T-cell therapy, raise concern for bridging therapy for disease stabilization prior to CAR T-cell therapy administration, as well as subsequent therapies [63]. 

### 2.3. Multiple Myeloma

Multiple myeloma (MM) accounts for ~10% of all hematologic malignancies in the United States, with the highest incidences observed in developed countries, and considered to be a non-curable disease with inevitable relapse [64]. In contrast to previous two B-cell malignancies, myeloma cells rarely express CD19. Identifying other targets thus disadvantages the application of CAR T-cell therapy in MM. Recently, a member of the TNF receptor superfamily, B-cell maturation antigen (BCMA), which binds to B-cell activating factor and a proliferation-inducing ligand (APRIL) [65,66], was found to express on primary human CD34+ hematopoietic cells and are commonly expressed in plasma cells and primary myeloma cells [67,68]. This then became the target of CAR T-cell therapy in MM [69,70,71]. Currently, multiple BCMA CAR T-cell products are under investigation, including the first phase III study, KarMMa-3 study (NCT03651128). These compared the bb2121 BCMA CAR T-cell product to the current standard regimen of daratumumab-containing triplet therapies, in patients previously receiving more than 2 lines of therapy. A phase II study of LCAR-B38M (NCT03758417), a phase IB/II study of JNJ-68284528, namely CARTITUDE-1 study (NCT03548207) and a phase I/II study of JCARH125 (NCT03430011) referred to as the EVOLVE study is also ongoing. The updated analysis for these trials showed ORR  >  80% in patients with r/r MM [72]. However, the duration of response ranged from 4 to 16 months [69,73] and the details of this study are subject to further scrutiny. The BCMA CAR T-cell therapy is awaiting approval for treatment of r/r MM with more than three prior lines of therapy. Nevertheless, earlier intervention of CAR T-cell therapy in the disease course was investigated in the KarMMa-2 phase II study (NCT03601078). Other strategies to improve persistence and efficacy of the BCMA CAR T-cell therapy included dual CAR T-cell therapy targets of both CD19 and BCMA [28,74], combination of infusion components [75,76], altered construct [77,78,79], and enriched culture medium [80,81].

### 2.4. Other r/r Lymphocytic Disease

In r/r chronic lymphocytic leukemia, the response rate was around 50% using CD19 CAR T-cells therapy [82,83], and the rate even lowered to 30% once the relapse happened post HST [84]. This diminished effect in CLL could be due to the expression of different phenotypes of CD4+ T-cells. Specifically, the CLL patients were found to display less “naïve” CD4+ T-cells, which is critical for CAR T-cell persistence. In addition, the naïve CD4+ T-cells of CLL patients express even more exhaustion markers [85], such as strong expression of PD-1, CD160, and CD244, and their CD8+ T-cells have low proliferative and cytotoxic capacities [86]. These intrinsic characteristics are indeed favored by previous lines of treatment (with fludarabine, in particular), but frustrate the use of CAR T-cell therapy. Further management, including improved long-term expansion and maintenance of CAR T-cell populations using Ibrutinib, which redirects the immune response of autologous T-cells from a Th2 profile to a Th1 profile will be evaluated in clinical trials (NCT03331198) [87]. The next stage of CAR T-cell therapy for lymphoblastic leukemia could be targeting the CD30 antigen for Hodgkin lymphoma (HL), and clinical trials are underway [35,88].

### 2.5. R/r Myeloid Leukemia

The main obstacle of application of CAR T-cell therapy to myeloid malignancies lies in the lack of unique target antigens that is distinct in malignant cells from healthy progenitor cells. For a long time, a number of target antigens were proposed, including CD123 [89,90], LeY antigen [91], folate receptor-β [92], and CD33 [93], based on preclinical studies. Other antigens like FLT3, CD7, ADGRE2, CCR1, CD70, and LILRB2 are summarized in Cummins and Gill’s work [94]. Sporadic case reports are congruous with the promising data of CD123- and CD33-directed CAR T-cell therapy in pre-clinical models [93,95,96], with more than 20 clinical trials ongoing [97], and we listed all phase II/III CAR T-cell trials containing adult r/r acute myeloid leukemia (AML) in Table 2. To avoid the off-target killing of normal hematopoietic cells, limited CAR T-cell persistence, in contrast to B-cell malignancies, might circumvent neutropenic infections and bleeding complications [97]. Multiple studies on controlling CAR T-cell persistence were carried out, including engineering a suicide gene in CAR T-cell, such as herpes simplex virus-thymidine kinase (HSV-tk) [98] or inducible caspase 9 (iCasp9) [99,100]. These co-expressed a well-characterized surface antigen, whose monoclonal antibodies are available [101,102], or used a less persistence costimulatory domain of CD28 rather than high persistent 4-1BB [103]. In addition, blasts in AML, actually hampered the production of CAR T-cell [104], with only 1/3 patients showing sufficient numbers for further manufacture in a small phase I study involving CD123 CAR T-cell [105]. This would need further manifestations in engineering CAR T-cell.

## 3. The Toxicity and Limitation of CAR T-Cell Therapy

### 3.1. Toxicity

Although CAR T-cell therapy represents a relatively new era of treatment, its two major adverse effects, namely cytokine release syndrome (CRS) and neurotoxicity were reconcilable along trials [106,107], which could be severe or fatal, if unrecognized. Here, we highlight the significance of close monitoring and early appropriate interventions.

#### 3.1.1. CRS

Despite the life-saving result of CAR T-cell in clinical trials, its severe toxicities could also be life-threatening [108]. The most widespread severe toxicity, CRS, emanated from inflammatory interaction between CAR T-cells and tumoral B-cells. Once they were activated and expanded, cytokines, mainly IFN-γ and TNF-α, were released from cell lysis. Moreover, the tumoricidal activity of monocytes and macrophages were magnified and emancipated high levels of pro-inflammatory cytokines, including IL-6, IL-1, and IL-10 [109,110,111]. Symptoms of CRS ranged from high fevers, refractory hypotension, tachycardia, hypoxia, consumptive coagulopathy, and multiple end-organ failure [111,112]. One out of four patients present severe CRS [110,111]. It was reported that B-ALL had as high as 29.3% server CRS, compared to those with B-lymphoma (19.8%) [113]. Risk factors could be derived from three main aspects—patient-related factors such as infection or inflammatory state, as well as hematopoietic cell transplantation-comorbidity index (HCT-CI), tumor-related factors, including disease type and tumor burden, and CAR T-cell related factors, that is CAR T-cell design and product expansion number. In addition, conditioning chemotherapy containing fludarabine was associated with the development of severe CRS [113]. Recent American Society for transplantation and cellular therapy has consensus on grading CRS according to body temperature, hypotension requiring a vasopressor or not, and oxygen demand from grade 1 to 4 [114,115]. Management of toxicity was based on its clinical presentations, and tocilizumab, an anti-interleukin (IL)-6 receptor antibody, was suggested once toxicity reached grade 2 or higher [114].

#### 3.1.2. Neurotoxicity

Neurotoxicity is the second most common toxicity in CAR T-cell therapy, also referred to as immune effector cell-associated neurotoxicity syndrome (ICANS) or CAR T-cell-related encephalopathy syndrome (CRES). It contains diverse symptoms that are not limited to one region of body, such as headache, delirium, hallucinations, cognitive defects, expressive aphasia, apraxia, somnolence, tremors, ataxia, nerve palsies, focal motor or sensory deficits, myoclonus, etc. It can also progress to severe encephalopathy, including seizures, obtundation, and even cerebral edema, which leads to death [116]. It was proposed that there are two patterns of neurotoxicity [108]. One might correspond to breakdown of the blood–brain barrier through cytokine production of IL-1, IL-6, and TNF-α, which occur immediately after CRS and affect angiotensin 1/2 balance, bringing stress to brain vascular pericyte and enhancing endothelium-activating cytokines [117]. The other pattern is linked to the expansion and activation of CAR T-cells, which lead to a direct parenchymal CAR T-cell infiltration into central nervous system, where pan-T encephalitis was demonstrated in an animal model [118]. This finding correlates with the finding that higher serum level of inflammatory markers such as CRP, early peak of IL-6, IL-2, sIL-2Rα, IL-6, IL-8, IL-10, IL-15, INF-γ, TNF- α, granzyme B, soluble GM-CSF, and MCP-1 [106], as well as elevated protein and multiple cytokines in the cerebrospinal fluid (CSF), accompany severe neurological toxicity [119,120]. Although there is no clear link between the costimulatory domain and neurological toxicity, anti-CD22 CAR T-cell for ALL exhibited a favorable neurotoxicity profile compared to that of anti-CD19 [23], and anti-BCMA displayed less frequent severe neurological toxicity [69,121]. Further clinical trials containing a larger number of samples are needed to define the correlation. While the pathogenesis of neurological toxicity remains unclear, its toxicity grade was observed to be associated with higher grade CRS [41,112,119], revealing overlapping risk factors with CRS, despite independent mechanisms. The severity could be graded using Common Terminology Criteria for Adverse Events (CTCAE) system, which was widely used across many centers [106]. The CAR-T-cell therapy-associated (CARTOX) consensus group also published a grading system for CAR T-cell neurological toxicity, which mainly focuses on cognitive functions, except that seizures, motor weakness, or papilledema directly result in grade of 3 or higher [111]. The Pediatric Oncology Group at the NCI and colleagues developed their own grading system combining patients’ cognitive test and observer-reported checklist [122]. The recent ASTCT consensus grading system then highlights the immune effector cell-associated encephalopathy scores, seizures, motor weakness, and raised intracranial pressure or cerebral edema as a new index [114]. It is suggested to closely monitor neurological toxicity throughout the treatment and exclude other possible factors like infection or electrolyte imbalance, which leads to neurotoxicity. Levetiracetam 500 mg twice daily for seizure prophylaxis is also recommended to use in patient with grade 1–2 neurotoxicity and steroid in grade 2–3 [114].

### 3.2. Limitations

CAR T-cell therapy, which replaces “drug” with “cell”, encompasses several advantages as well as disadvantages. Its advantages in hematological malignancies include constant contact of CAR T-cell with malignant cells. Additionally, patients could be easily followed up through peripheral blood draw. The ceaseless contiguity is indeed one of its disadvantages, as the inflammatory response and cytokine milieu might also stimulate malignant cells. Additionally, as malignant cells originate from normal immune system and hemopoietic cells, in patients receiving CAR T-cell, it would display server deficient immune system and neutropenia, which are susceptible to infections [123]. Importantly, the close exposure might lead to easy immune evasion, thus reducing CAR T-cell potency [124]. Finally, as CAR T-cell is a highly personalized medicine, its cost is still high and did not yet reach general commercial production. Additionally, as CAR T-cell therapy is currently used in r/r disease, the prior treatments and conditioning therapies differs, further effort is needed to reach a consensus.

### 3.3. Challenges

The pioneering work in CAR T-cell therapy is altering the treatment for patients with r/r hematological malignancies. However, despite the current remarkable results, many challenges remain in this field. First, post-CAR T-cell relapse was reported and is becoming more of a concern as CAR T-cell therapy is more widely used. There are two main patterns of post-CAR T-cell relapse—one is evading CAR-mediated recognition and clearance because of the lack of targeting antigen, whereas the other is the lack of CAR T-cell persistence [125]. The former could be overcome by using a combination of either another CAR T-cell, or another monoclonal antibody. Indeed, CD22 CAR T-cell was used in post-CD19 CAR T-cell relapse [23], and a dual CAR T-cell, which targeted both CD19 and CD20 or CD22 was proposed [31]. Other monoclonal antibodies, such as rituximab and inotuzumab, could both be combined with non-CD20 or non-CD22 CAR T-cell therapy to widen targets range.

The later could also be improved on multiple aspects. First, by modifying the CAR structure, such as replacing murine scFv with humanized scFv, and using a 4-1BB costimulatory molecule instead of CD28 [125]. CAR gene editing using CRISPR/Cas9 technology to “knock-in” a designated gene locus [126] as well as “knocking-out” inhibitory receptors, such as LAG-3, TIM-3, and CTLA-4 [127] (which were found to be highly expressed when T-cells are exhausted from chronic activation [128,129]) or CD3, HLA-I, Fas triple-ablated CAR T-cells in vitro and in vivo, also ameliorate CAR T-cell efficacy by enhancing the anti-tumor activity of T-cells [130]. Designing artificial antigen-presenting cells that could activate CAR T-cells by releasing IL-21 and IL-15 was also able to stimulate and amplify the number of CAR T-cells [131,132]. The use of checkpoint inhibitors, such as nivolumab, atezolizumab, or durvalumab [133,134,135], by regulating the programmed death ligand 1/programmed cell death 1 (PD-L1/PD-1) axis, also enhanced the anti-tumor activity of T-cells [136] to modify tumor microenvironment [137]. Other “consolidation” therapy, such as Lenalidomide (NCT03070327) [138], cereblon-modulating agent CC-122 (NCT03310619), celecoxib, and histone deacetylase inhibitors as possible modalities [139], are under investigations. In addition, whether it is advisable to receive hematopoietic cell transplant (HCT) following CAR T-cell therapy, is still under debate [140]. Recent report indicates that HCT appears to benefit subjects that attain a complete response but are at an increased risk of relapse [141].

The second issue would be to generate “off-the-shelf” immunotherapy in the hopes of reducing the risk of graft-versus-host disease. Recently, Boissel et al. [142] successfully produced CD19-CAR NK cells from the NK-92 cell line, with cytolytic function against resistant chronic lymphoblastic leukemia cells [143]. Efficient CD19-CAR NK cells against B cell leukemia [144,145,146] and myeloma [147] were also reported. “Armored CAR”, possessing the ability to secrete cytokines like IL-18, which constitutively express CD40L to enhance IL-12 secretion or constitutively express 4-IBB ligand to modulate the tumor microenvironment, are also being examined [148,149]. Additionally, targeting extracellular matrix or T-lymphocyte exclusion pathways, e.g., VEGF or TGFβ, which are future avenues for applications of CAR T-cell in solid tumors, might in turn provide a possible method to modify the tumor microenvironment in hematological malignancies [150].

In addition to the basic science, a couple of issues regarding CAR T-cell therapy applied in current practice should be taken into considerations as well. As the aforementioned CAR T-cell trials were all employed in r/r hematological malignancies, it would be of great interest to study if earlier applications of CAR T-cell therapy would benefit the outcome, which, in particular, would change the treatment paradigm of hematological cancer. In addition, the initial CAR T-cell therapy got striking success in young adults, whereas most hematological malignancies, such as myeloma, were diagnosed in people older than 60 years old. In the elderly, the frailty of the patient should be taken into considerations [151], and might need close surveillances. Finally, as CAR T-cell therapy endures highly personalized medicine, questions about dosing, timing, and duration of response requires well-designed clinical trials to answer. At this point, current data are too trivial to reach a conclusion, but expanding experiences outside of single centers is growing. From the financial point of view, a structured manufacturing process should be reached to lower the cost, and quality of production should be regulated as more and more companies step into the field [152,153]. With the understanding of the molecular and physiology of this therapy, and strong cooperation between the laboratory and the clinic, CAR T-cells are anticipated to be used in an increasing number of patients with hematological malignancies and would shape the future of this field dramatically.

## Figures and Tables

**Table 1:** Current CAR T-cell therapy targets in hematological malignancies.

Disease	CAR T-Cell Therapy Targets	Phase
Acute Lymphoblastic Leukemia	CD19 [21,22] CD22 [23,24] Dual CD19 and CD22 [25] Dual CD28 and CD137 (NCT02186860) CD33 (NCT02799680) CD20/CD22/CD10 (NCT03407859) CD33/CD38/CD56/CD123/CD117/CD133/CD34/Mucl (NCT03473457) CD123 (NCT03556982) CD38 (NCT03754764) CD7 (NCT04004637) 4SCAR-CD22/CD123/CD38/CD10/CD20/TSLPR (NCT04016129) XYF19 (NCT04037566)	I, II I, I I I I I N/A I/II I/II I I/II I
Multiple Myeloma	BCMA ^1^ [26,27] BCMA and/or CD19 [28] CD138 (NCT01886976) CD138/BCMA/CD19/more (NCT03196414) BCMA/CD38/CD56/CD138/alternative antigens (NCT03271632, NCT03473496) CD38 (NCT03464916) NY-ESO-1 (NCT03638206) IM21 (NCT03711864, NCT04537442) Dual BCMA and CD38 (NCT03767751) Integrin β7/BCMA/CS1/CD38/CD138 (NCT03778346) CD44v6ΔNL (NCT04097301) CD4 (NCT04162340) SLAMF7 (NCT04499339) CS1 (NCT04541368)	Ib/II, I I/II I/II I/II I/II, N/A I I/II I, I I/II I I/II I I/II I
Diffuse Large B Cell Lymphoma	CD19 [29,30] Dual CD19 and CD20/CD22 [31]	II, II In vivo
Non-Hodgkin Lymphoma	CD19 [21,32,33] Dual CD19 and CD20 [34] CD19/CD20/CD22/CD30 (NCT03196830) CD20 (NCT03664635, NCT04169932) Dual CD19 and CD22 (NCT04303247, NCT04412174)	I, I, I I II I/II, I I, I
Hodgkin Lymphoma	CD30 [35]	I
NK/T-Cell Lymphoma T-Lymphoblastic Lymphoma	CD7 (NCT04004637, NCT04572308)	I, N/A
Acute Myeloid Leukemia NK Cell Lymphoma T-Cell Acute Lymphoblastic Leukemia	CD7 (NCT04033302)	I/II
Adult T-Cell Lymphoma/Leukemia Anaplastic Large Cell Lymphoma Angioimmunoblastic T-Cell Lymphoma Hodgkin Lymphoma NK/T-Cell Lymphoma Peripheral T-Cell Lymphoma	CD30 (NCT04008394)	I
Anaplastic Large Cell Lymphoma Extra-nodal NK/T-Cell Lymphoma Diffuse Large B Cell Lymphoma Peripheral T-Cell Lymphoma Primary Mediastinal Large B-Cell Lymphoma	CD30 (NCT04526834)	I
Lymphoma	CD30(NCT02259556, NCT02917083) 4SCAR20/22/70/PSMA/13/79b/GD2 (NCT04429438)	I/II, I I/II
Chronic Lymphocytic Leukemia	CD19 (NCT03085173)	I
Acute Myeloid Leukemia	CD33 [36] CD123 [37] CLL-1+CD33 [38] Muc1/CLL1/CD33/CD38/CD56/CD123 (NCT03222674) CD38/CD33/CD56/CD123/CD117/CD133/CD34/Mucl (NCT03473457) CD123/CLL1 (NCT03631576) CLL-1/CD33 and/or CD123 (NCT04010877) CD44v6ΔNL (NCT04097301) CD19 (NCT04257175) CD38 (NCT04351022)	I/II I I N/A N/A I/II N/A II/III II/III I/II
Acute Myeloid Leukemia Myelodysplastic Syndrome	NKG2D (NCT03018405) CD33/CD38/CD56/CD117/CD123/CD34/Muc1 CAR T-cells + Eps8 or WT1 peptide specific dendritic cell (NCT03291444)	I/II I
Acute Myeloid Leukemia Myelodysplastic Syndrome Myeloproliferative neoplasms	CLL1-CD33 (NCT03795779) CD123-CD33 (NCT04156256)	I I
Chronic Myeloid Leukemia	IL-1RAP (NCT02842320)	N/A
CD4+ T-Cell Lymphoma	LCAR-T2C (NCT04219319)	I

The order of the diseases listed in the table are arranged according to the order mentioned in the main text. In each disease, antigens with known publication are listed in Figure 1. ^1^ BCMA (B cell maturation antigen).

**Table 2:** Current CAR T-cell phase II/III clinical trials including adult relapsed/refractory acute myeloid leukemia.

Clinical Trial and Institution	Target	Phase	N	Age	Lymphodepleting Chemotherapy
NCT04033302 Shenzhen Geno-Immune Medical Institute	CD7	1 & 2	30	6 Months to 75 Years	Not mentioned
NCT02742727 PersonGen BioTherapeutics (Suzhou) Co., Ltd.	CD7	1 & 2	10	18 Years and older	Not mentioned
NCT04257175 Sheba Medical Center	CD19	2 & 3	10	18 Years and older	cyclophosphamide and fludarabine
NCT03896854 Shanghai Unicar-Therapy Bio-medicine Technology Co., Ltd.	CD19	1 & 2	15	6 Years to 65 Years	Not mentioned
NCT03971799 Center for International Blood and Marrow Transplant Research	CD33	1 & 2	34	1 Year to 35 Years	cyclophosphamide and fludarabine
NCT01864902 Chinese PLA General Hospital	CD33	1 & 2	10	5 Years to 90 Years	Not mentioned
NCT04351022 The First Affiliated Hospital of Soochow University	CD38	1 & 2	20	6 Years to 65 Years	Not mentioned
NCT04097301 MolMed S.p.A.	CD44v6	1 & 2	58	1 Year to 75 Years	cyclophosphamide and fludarabine
NCT03556982 The Affiliated Hospital of the Chinese Academy of Military Medical Sciences	CD123	1 & 2	10	14 Years to 75 Years	Not mentioned
NCT04272125 Chongqing Precision Biotech Co., Ltd.	CD123	1 & 2	40	3 Years to 75 Years	Not mentioned
NCT04265963 Chongqing Precision Biotech Co., Ltd.	CD123	1 & 2	45	2 Years to 75 Years	Not mentioned
NCT04109482 Mustang Bio	CD123	1 & 2	126	18 Years and older	Cyclophosphamide, fludarabine and decitabine
NCT03631576 Fujian Medical University	CD123/CLL1	2 & 3	20	up to 70 Years	Not mentioned
NCT04010877 Shenzhen Geno-Immune Medical Institute	CD33, CD123/CLL-1	1 & 2	10	6 Months to 75 Years	Not mentioned
NCT03222674 Shenzhen Geno-Immune Medical Institute	Muc1/CLL1/CD33/CD38/CD56/CD123	1 & 2	10	2 Years to 75 Years	Not mentioned

The table enlightening current CAR T-cell phase II/III clinical trials targeting relapsed/refractory acute myeloid leukemia in adults, arranged according to the CAR T-cell target, from a small to a large number.
